# Management of Cerebral Herniation Secondary to Lead Encephalopathy: A Case Report

**DOI:** 10.3389/fneur.2022.893767

**Published:** 2022-05-20

**Authors:** Somnath Das, Felicia Hataway, Hunter S. Boudreau, Yasaman Alam, Jordan A. George, William Rushton, Sukhshant Atti, Manmeet Kaur, Marshall T. Holland

**Affiliations:** ^1^Department of Neurosurgery, University of Alabama Birmingham, Birmingham, AL, United States; ^2^Department of Neurology, University of Alabama Birmingham, Birmingham, AL, United States; ^3^School of Medicine, University of Alabama Birmingham, Birmingham, AL, United States; ^4^Office of Toxicology, University of Alabama Birmingham, Birmingham, AL, United States

**Keywords:** lead encephalopathy, intracranial pressure, ventriculostomy, lead toxicity, neurocritical care

## Abstract

**Background:**

Adult lead encephalopathy is a rare but critical condition to recognize in modern healthcare settings. Few reports have described the medical and neurosurgical management of severe adult lead encephalopathy.

**Case Presentation:**

A 22 year old woman presented with severe headache, anemia, vomiting, 40-lb weight loss, and constipation. At the time of presentation, she had extensive colonic radiopaque material and a serum lead concentration of 87 mcg/dl (normal <10). She rapidly developed anisocoria requiring emergent ventriculostomy insertion. Following CSF diversion, ICP mitigation, and lead chelation, she considerably improved in <2 weeks.

**Conclusion:**

We report one of the few instances of successful surgical and medical management of adult lead encephalopathy. Dedicated neurocritical care and neurosurgical teams are necessary in conjunction with toxicology in order to manage the advanced sequalae of severe lead poisoning.

## Introduction

Improved late twentieth century regulations on use of lead in common products such as household paints and petrochemicals has reduced the cumulative lead exposure in the U.S. population (1); however, lead poisoning is still a significant public health issue that risks going unrecognized in modern healthcare settings. Encephalopathy is a feared complication of lead exposure. Lead can cross the blood-brain barrier and can cause neuronal dysfunction at both low and high concentrations (2).

Given unique vulnerabilities, the pediatric population is at greatest risk of developing lead encephalopathy leading to cerebral edema. Reports of lead encephalopathy in the adult population are increasingly rare; one of the largest case series was published from our institution approximately 30 years ago (3). Moreover, there is a paucity of literature reporting the management of elevated intracranial pressure secondary to lead encephalopathy, with only two prior case reports reporting the use of ventriculostomy (4, 5). The report herein describes a case of advanced lead encephalopathy in a 22-year old woman requiring cerebrospinal fluid (CSF) diversion and continued intracranial pressure (ICP) management. The patient demonstrated both clinical and radiological improvement in their cerebral edema, prompting subsequent removal of her ventriculostomy.

## Case

**Figure 1:**
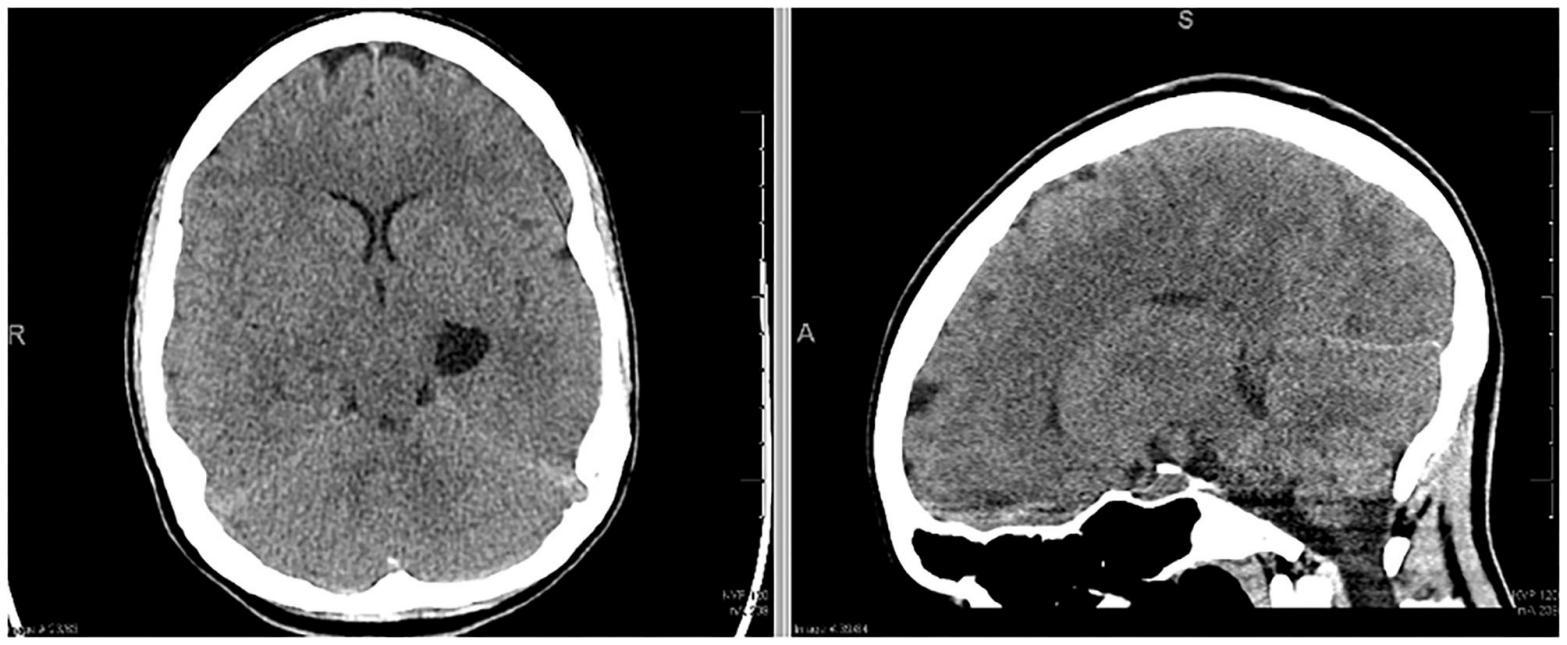
Initial non-contrast CT of the head demonstrating left temporal cyst.

A 22 year-old woman presented to the emergency department with generalized feelings of weakness, vomiting, abdominal pain, constipation, a 40-lb weight loss, and 2 days of intermittent, severe headache. She had undergone a prior workup over the previous 3 months for self-reported syncopal episodes, abdominal pain, vomiting and chronic constipation. In the 2 weeks leading up to her hospitalization, her mother also reported a history of episodic right sided weakness and right-sided facial droop. Initial non-contrast computed tomography (CT) scan (Figure 1) demonstrated a left sided temporal cyst. An MRI obtained at the time demonstrated that this cyst had a component of diffusion restriction. In addition, there were subtle FLAIR hyperintensities in the bilateral parietal and occipital lobes that thought to be secondary to leptomeningeal enhancement (Supplementary Figure 2). No cerebral edema was visualized on CT imaging. While in the emergency room, she had a witnessed tonic-clonic seizure aborted by diazepam, followed by a prolonged post-ictal period of ~4 h. She developed a temperature of 38.9 degrees Celsius and became increasingly encephalopathic after the seizure, prompting a lumbar puncture and empiric meningitis treatment. Her opening pressure was >55 cm H_2_O with an initial CSF profile of glucose 80 mg/dl, protein 10, WBC 10 × 10^9^/L, RBC 525 × 10^9^/L. Her neurological exam at the time of lumbar puncture did not reveal any focal deficits. Given her recent imaging, exam, and CSF profile, she was started on empiric treatment and workup for encephalitis.

**Figure 2:**
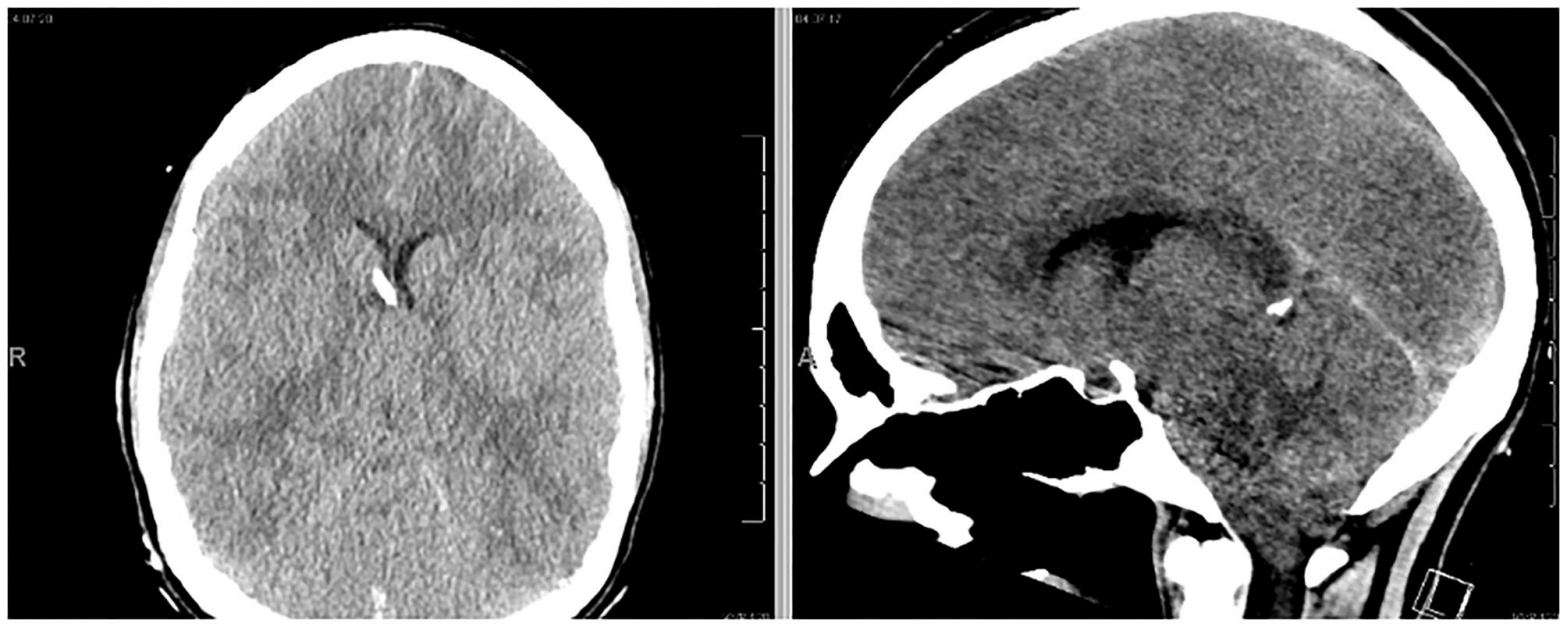
The patient exhibited anisocoria and severe headache following hospital admission. Follow-up CT scan demonstrated profound cerebral edema and downward tonsillar herniation. An EVD was successfully placed (left image).

Approximately 18 h after her lumbar puncture, the patient became anisocoric. She reported a severe headache but demonstrated intact motor strength and sensation of bilateral upper and lower extremities. A second emergent CT scan of the head was obtained, demonstrating downward cerebral tonsillar herniation and global edema (Figure 2). Following 3% hypertonic saline bolus and admission to the neuro-intensive care unit (NICU), an external ventricular drain (EVD) was placed successfully, with an opening pressure of 58 mm Hg. The patient was subsequently intubated for airway protection as the patient was increasingly unable to clear secretions. ICP management was initiated with sedation (propofol, fentanyl), hypertonic therapy, acetazolamide 1 gm BID, and dexamethasone. Midazolam was added due to intermittent ICP spikes into the 30 s. Extensive testing for autoimmune and infectious sources of her presentation were initiated and unrevealing. It was later endorsed by a patient's family member that the patient had been taking “supplements” for months leading up to admission; the toxicology team was consulted. As part of the workup, abdominal x-ray was obtained which showed evidence of radio-opaque material (Supplementary Figure 1), which prompted workup for heavy metal poisoning.

The following day, a peripheral blood smear demonstrated basophilic stippling, and thus the patient was empirically started on dual lead chelation therapy with oral Succimer 10 mg/kg TID and intramuscular Dimercaprol (BAL: British Anti-Lewisite) 4 mg/kg QID. The patient was also placed on hematin therapy due to a potential diagnosis of acute intermittent porphyria; this was later discontinued to lead being the likely etiology of her elevated urinary porphyrins. Calcium disodium ethylenediaminetetraacetic acid (CaNa_2_EDTA), typically used in combination with BAL to treat lead encephalopathy, was not available due to a nationwide shortage. We initiated aggressive whole bowel irrigation to remove potential further lead exposure from her intestines (6). Her serum lead concentration resulted at 87 mg/dl; other heavy metals (arsenic, antimony, selenium, mercury, thallium) were undetectable.

**Figure 3:**
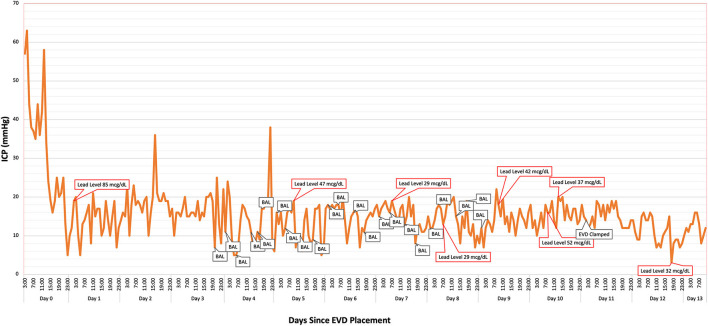
Intracranial pressure (ICP) was monitored continuously *via* the patient's ventriculostomy. British anti-Lewsite (BAL: dimercaprol) injection times likely corresponded to intermittent elevations in ICP. Optimal pain sedation with hydromorphone, midazolam, and lidocaine helped mitigate the severity of the spikes. A steady decline in the patient's lead levels corresponded to improvement in her cerebral edema and ICP. Lead concentration was 14 mcg/dl at 5 days post-EVD removal.

The patient experienced multiple ICP spikes following initiation of BAL chelation therapy secondary to the painful nature of the injections. Due to sustained ICPs > 35 mmHg, the patient was started on a cisatracurium drip. A pain protocol of lidocaine 2% intramuscularly at the injection site prior to BAL administration followed by intravenous hydromorphone (2 mg) after injection reduced the severity of ICP spikes over time (Figure 3). The patient developed mild transaminitis and hypertriglyceridemia mildly concerning for propofol infusion syndrome. Propofol was weaned off and we maintained the same level of sedation with fentanyl and midazolam (as per EEG) while on concomitant paralytic therapy. Due to the patient's concurrent lead-induced porphyria, pentobarbital therapy was deferred.

**Figure 4:**
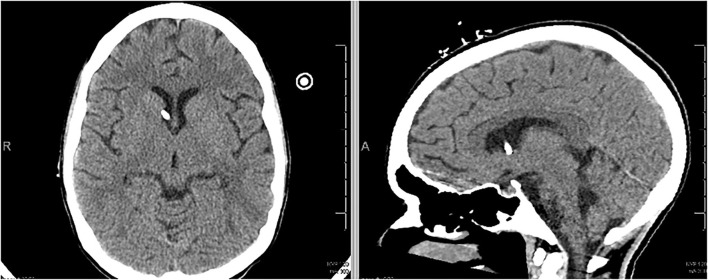
Non-contrast CT scan demonstrating improved cerebral edema on day of EVD removal.

The patient underwent a total of 19 days of chelation therapy combined with both whole bowel irrigation and endoscopic washout with gastroenterology. Her ICPs stabilized and serum lead concentrations continued to decrease. Repeat surveillance imaging was obtained at 12 days post-EVD insertion, which demonstrated marked improvement in her cerebral edema. The EVD was then removed at 13 days post-insertion (Figure 4). Serum lead concentration was 14 mcg/dl at 5 days post-EVD removal. She was extubated and ultimately failed the trial due to issues with clearing secretions. She underwent tracheostomy placement and was subsequently transferred to a pulmonary special care unit. Despite improvements in her mentation, she had an increase in her measured blood lead level to 44 mcg/dl—likely secondary to redistribution of chronic store of lead in the bony matrix (7). She was restarted on chelation therapy. Upon discharge, she was decannulated from her tracheostomy and had a non-focal neurological exam. She is continuing an aggressive bowel regimen and lead chelation therapy with Succimer 10 mg/kg BID as an outpatient. Her most recent blood lead level upon discharge was 26 mcg/dl.

## Discussion

Lead is a potent neurotoxin, which with acute and chronic exposure, can contribute to cortical atrophy and poor neurocognitive outcomes (8). Intracranially, lead can cause widespread lesions at the gray-white junction leading to FLAIR hyperintensities as seen in our patient and prior case reports (9). In addition, lead encephalopathy can cause cystic lesions that could be confounded for a mass (10, 11), which was also observed in this case. Lead readily crosses the blood-brain barrier due to its ability to mimic the function of calcium ions (Ca^2+^) and via its affinity for Ca-ATPase pumps along brain capillary endothelial cells, allowing its back transport into the brain (12). Neuronal lead accumulation mimics intracellular calcium accumulation which triggers mitochondrial transition pore dysfunction, leading to eventual neuronal apoptosis and resultant cerebral edema. In addition, lead disrupts synaptic transmission of multiple neurotransmitters (2). The resultant encephalopathy and cerebral edema is life threatening if left unrecognized.

While more commonly reported in children, adult lead encephalopathy remains a rare but critical phenomenon. Prior to Whitfield et al.'s case series in 1972, it was estimated that only 31 patients with lead encephalopathy were reported in English literature. Whitfield et al. reported 23 adults with lead encephalopathy at the University of Alabama Birmingham over 10 years. Most patients had seizures (82%) and anemia (90%). Three patients expired due to cerebral edema, and one patient had bilateral uncal and tonsillar herniation. Seventy-eight percent of patients had a favorable response to CaNa_2_EDTA therapy (3). Symptoms of severe lead encephalopathy (+/– serum lead concentration >100 mcg/dl) have traditionally been treated using calcium disodium ethylenediaminetetraacetic acid (CaNa_2_EDTA) with concomitant BAL therapy given the combination therapy's efficacy to decrease serum lead concentration to 50% or less within 15 h (13). We were unable to procure CaNa_2_EDTA given a national shortage. Thus, we opted for combination BAL and Succimer therapy to manage our patient, which was efficacious in decreasing patient's serum lead concentration quickly.

There are few descriptions of surgical management of severe lead encephalopathy. Harrington et al. report a 3-year-old female who presented after 2 months of lead paint pica who had a mass in her cerebellar vermis causing cerebellar tonsil herniation. Following EVD insertion, suboccipital craniectomy, and lead chelation, the child's intracranial pressures normalized and the mass resolved. Pathological specimens from their surgery were consistent with edematous cerebellar tissue (5). Berkowitz and Tarrago (4) report a 4-year-old male who initially presented with vomiting and sleepiness that progressed to obtundation. The patient required ventriculostomy, and unfortunately died soon after. The patient did not receive chelation therapy due to the quick progression of his symptoms. Talbot et al. report a 2-year-old boy who presented with seizures and 2 weeks of vomiting and was started on CaNa_2_EDTA chelation therapy. Despite a reduction in blood lead levels, the patient's neurological status deteriorated, and care was withdrawn (1). Thus, in all cases of severe lead encephalopathy, the possibility of an elevated ICP should be considered. Based on our experience, the urgent removal of the source of exposure, dual lead chelation therapy and ICP mitigation may all be necessary in order to avert sequelae of cerebral edema secondary to lead encephalopathy. An awareness of the adverse effects of chelation therapy is also vital toward proper management of severe lead encephalopathy.

## Conclusion

We present one of the few reports of successful neurosurgical and medical management of severe lead encephalopathy in an adult patient where successful treatment of an elevated intracranial pressure substantially improved this patient's outcome. The patient required emergent CSF diversion followed by continued ICP monitoring and management. The diagnosis of acute lead encephalopathy was made based on based on a constellation of clinical signs, symptoms and diagnostic findings. In contrast to recommended standard of care, a combination of CaNa_2_EDTA and BAL were not utilized in our chelation regimen due to supply constraints. Our management strategy in the ICU consisted of dual chelation therapy utilizing BAL for 5 days and Succimer for 19 days, whole bowel irrigation, ICP mitigation (hyperosmolar therapy, sedation, paralytics), and CSF diversion. The patient demonstrated profound improvement in her cerebral edema and neurologic exam. Our results demonstrate the importance of the need for dedicated neurocritical and neurosurgical capabilities when managing severe lead encephalopathy.

## Data Availability Statement

The original contributions presented in the study are included in the article/Supplementary Material, further inquiries can be directed to the corresponding author/s.

## Ethics Statement

Ethical review and approval was not required for the study on human participants in accordance with the local legislation and institutional requirements. The patients/participants provided their written informed consent to participate in this study. Written informed consent was obtained from the individual(s) and/or minor(s)' legal guardian/next of kin for the publication of any potentially identifiable images or data included in this article.

## Author Contributions

SD: initial writeup. FH, YA, WR, SA, and MK: manuscript editing. HB and JG: figure design and manuscript editing. MH: project design, figure design, and manuscript editing. All authors contributed to the article and approved the submitted version.

## Conflict of Interest

The authors declare that the research was conducted in the absence of any commercial or financial relationships that could be construed as a potential conflict of interest.

## Publisher's Note

All claims expressed in this article are solely those of the authors and do not necessarily represent those of their affiliated organizations, or those of the publisher, the editors and the reviewers. Any product that may be evaluated in this article, or claim that may be made by its manufacturer, is not guaranteed or endorsed by the publisher.

## Supplementary Material

Supplementary Figure 1 Initial abdominal X-ray demonstrating copious radiopaque material.

Supplementary Figure 2 MRI demonstrating non-enhancing, diffusion restricting temporal cyst.
