# Supplementary figures and images for: Management of Cerebral Herniation Secondary to Lead Encephalopathy: A Case Report
Source: Front Neurol. 2022 May 20;13:893767. doi: 10.3389/fneur.2022.893767 (PMC9163400; doi:10.3389/fneur.2022.893767)

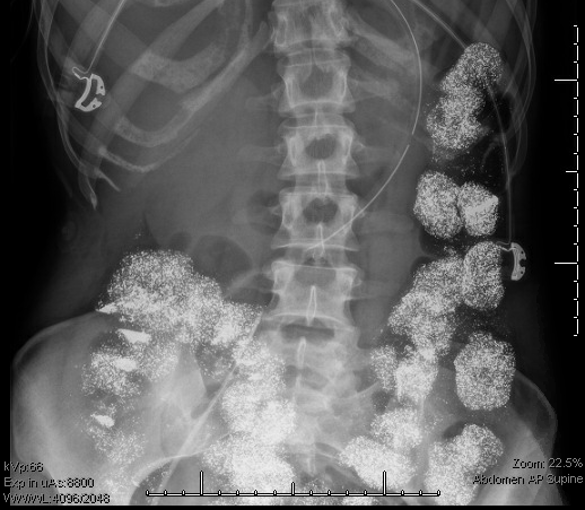

Supplement: Supplementary Figure 1 — Initial abdominal X-ray demonstrating copious radiopaque material. [file Image_1.TIFF]

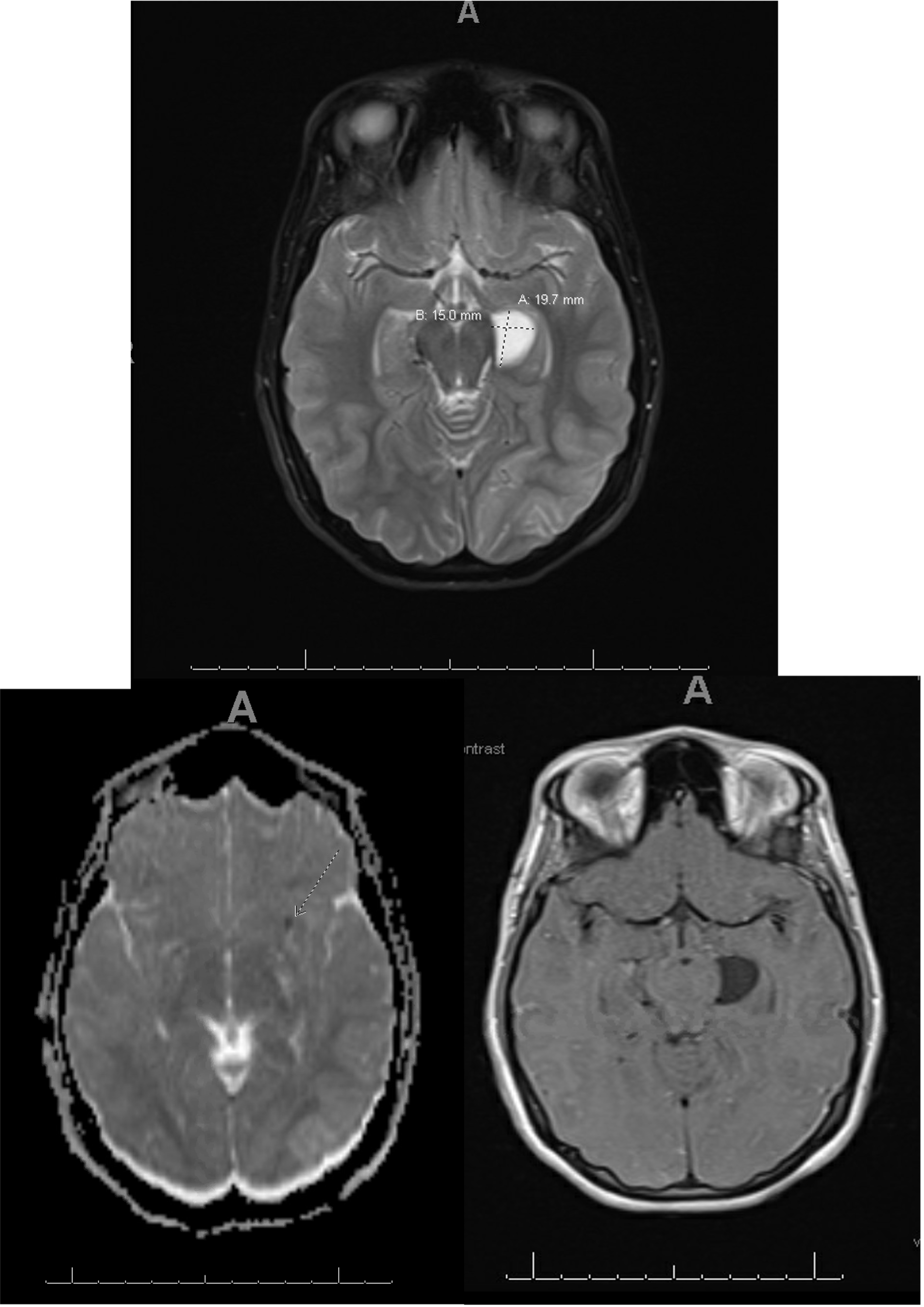

Supplement: Supplementary Figure 2 — MRI demonstrating non-enhancing, diffusion restricting temporal cyst. [file Image_2.TIFF]
